# Regulatory Mechanisms of Coicis Semen on Bionetwork of Liver Cancer Based on Network Pharmacology

**DOI:** 10.1155/2020/5860704

**Published:** 2020-11-21

**Authors:** Bonan Liu, Chen Bai

**Affiliations:** ^1^Key Laboratory of Carcinogenesis and Translational Research, Ministry of Education/Beijing, Department of Hepato-Pancreato-Biliary Surgery, Peking University Cancer Hospital & Institute, Beijing, China; ^2^Beijing University of Chinese Medicine, Beijing, China

## Abstract

At present, there is an increasing incidence and mortality of liver cancer. Despite surgery and chemoradiotherapy, there is a lack of effective oral medications with low side effects. In East Asia, Coicis Semen (CS) is used as both food and natural medicine and has a significant impact on the treatment of liver cancer. However, due to its multicomponent and multitarget characteristics, the mechanisms of CS against liver cancer remain unclear. This study collected CS compounds and target proteins in SymMap, then cross-matched with the liver cancer targets in the CTD database to construct an interaction network of CS-liver cancer proteins, and visualized by Cytoscape software. DAVID database was used to perform pathway enrichment analysis to find target proteins in core pathways and the related small molecules in CS. The results showed that a total of 103 common genes shared by CS and liver cancer were obtained, which were enriched for precancerous lesion pathways such as hepatitis B and fatty liver and biological signaling pathways such as HIF-1 and TNF. The combination of sitosterol and CASP3 in CS, acting on “pathways in cancer” and restoring normal cell apoptosis, could be the core mechanisms of CS in the treatment of liver cancer. Based on the system biology analysis, it is speculated that CS may not only participate in multiple mechanisms of action to treat liver cancer synergistically but may also be involved in factors that reduce the incidence of liver cancer.

## 1. Introduction

Liver cancer is the malignancy of the liver. According to the origin of the tumor, liver cancer can be divided into two types: primary liver cancer and secondary liver cancer. According to the latest statistics from the American Cancer Society [1], the incidence of liver cancer has continued to increase in recent years, with a 5-year relative survival rate of 18% after pancreatic cancer and increased mortality of liver cancer in the last 10 years. According to the statistics from the Chinese National Cancer for Cancer Prevention and Control [2], liver cancer is one of the four most common cancers diagnosed in China, and the most commonly diagnosed cancer among men less than 60 years of age, and is the leading cause of cancer death. Liver cancer is ranked 4^th^ in descending order among the most commonly diagnosed cancers in men. It is also the 4^th^ leading cause of cancer deaths. A report from Cancer Research UK has also pointed out [3] that liver cancer is expected to be one of the fastest-growing cancers by 2035, and the number of patients will increase significantly. Both local and global studies [4–6] have shown that the causes of liver cancer can be divided into four major categories: liver disease (such as hepatitis and liver fibrosis), bad living habits (drinking and obese diet), unhygienic diet (such as aflatoxin), and the unreasonable use of drugs (such as aristolochic acid).

New Clinical Practice Guidelines in Oncology (NCCN Guidelines®) have described the management of cancer by using therapeutic agents, surgical approaches, radiation therapy, and immunotherapy. Each treatment has its indications and contraindications, as well as unavoidable side effects. For example, the clinical significance of a commonly used drug sorafenib [7, 8] against liver cancer cannot be denied. Still, it may be accompanied by diarrhea, weight loss, skin toxicity, and drug resistance. Besides, the Drug database (http://www.drugs.com/) also includes more than 20 more common side effects, including abdominal pain, gingival bleeding, hematochezia, and more than 40 less common side effects. Therefore, it is urgent to find an effective, safe, and cheap drug, which can even intervene in clinical problems of liver cancer as a preventive measure.

Herbal medicines have been widely used in the treatment of liver cancer and other diseases as one of the complementary and alternative therapies due to their slow onset of action, multiple targets, and few side effects [9–11], which have gradually attracted people's attention. Coicis Semen (CS) is a dried and mature kernel of the Chinese herb Coix lacryma-jobi http://L.var.ma-yuen (Roman.). Its medicinal value was first recorded in the *Shennong's Materia Medica* before the Western Han Dynasty, at least 1800 years ago. According to *Chinese Pharmacopoeia-2015 Edition*, it can treat cancer-related clinical manifestations, including edema and cancer. At present, many studies have found that CS has a specific therapeutic effect on liver cancer from different aspects, as shown in Table 1 [12–20].

Current researches focus on the discussion of a particular pathway or mechanism. Due to complex chemical components and target proteins of plant-based natural medicines, it is difficult to analyze their complex mechanisms of action based on the classic pharmacology of molecular biology [11]. Network pharmacology can build a network of interactions between Chinese medicine compounds, targets, and diseases, which is conducive to revealing the complex mechanisms of action of multicomponent and multitarget medicines [21]. Therefore, this study explored the therapeutic mechanism of CS for liver cancer by network pharmacological approach.

## 2. Materials and Methods

**Figure 1:**
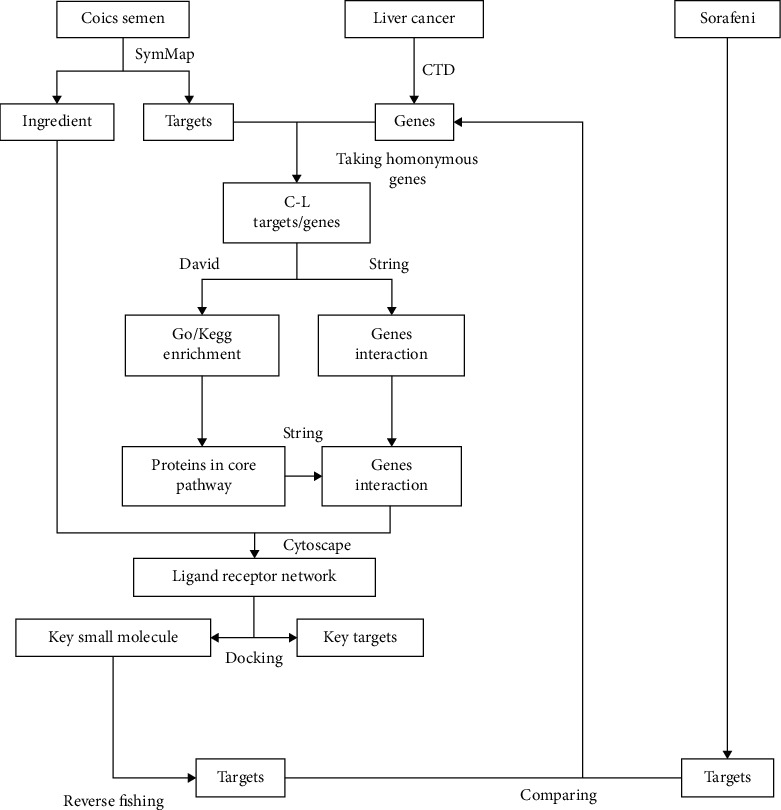
Flow chart of main research methods.

The methodology has been described in Figure 1.

### 2.1. Data Acquisition

In the SymMap database [22] (http://www.symmap.org/), click “Search,” enter “yiyiren” in the “Herb” tab, and click “search” to obtain “Ingredient” and “Target” in CS. In the CTD database (http://ctdbase.org/), select “Diseases,” enter “liver cancer,” and obtain “Genes” of “Carcinoma, Hepatocellular.” Use EXCEL software to get the Gene Symbol of CS Targets and Liver Cancer Genes with the same name.

### 2.2. Enrichment Analysis

In the David database (https://david.ncifcrf.gov/home.jsp), click “Start Analysis,” then enter the Gene Symbol of common target protein of “Coicis Semen-liver cancer” in the “Gene List,” and “Identifier” select “OFFICIAL_GENE_SYMBOL;” “Type” select “Gene List,” click “Submit List,” “species” select “Homo sapiens,” click “use;” select “GOTERM_BP_DIRECT,” “GOTERM_CC_DIRECT,” “GOTERM_MF_DIRECT,” and “KEGG_PATHWAY,” click “Functional Annotation Chart.” Bubble Chart was plotted using the OmicShare tools, a free online platform for data analysis (http://www.omicshare.com/tools).

### 2.3. Protein-Protein Interaction (PPI) Analysis

Using the String database as the background network database, the typical target genes of Coicis Semen-liver cancer and the target genes enriched to the core pathway were introduced, respectively. The research species was selected as “Homo sapiens,” and the target protein interaction was obtained and saved as a TSV file. Finally, TSV files were imported into Cytoscape 3.6 software to make network diagrams.

### 2.4. Construction of Protein-Molecular Relationship

In the SymMap database, the small molecules of “Coicis Semen” and the small molecules of “CASP3” were searched separately, and the small molecules shared by the two were extracted to construct a “Coicis Semen -small molecule-CASP3” protein relationship network.

### 2.5. Comparison of Effective Small Molecules in Coicis Semen with Sorafenib for Liver Cancer Targets Treatment

The molecular structures of “sitosterol” and “sorafenib” were obtained by using ChemDraw Professional software, and the 3D structural energy was minimized by using Chem3D software; saved as MOL2 format; and pharmmapper (http://www.lilab-ecust.cn/pharmmapper/) online tool [23–25] for reverse molecular docking; then clicked “Submit Job,” submitted the mol2 file in “Upload Query File;” clicked “continue,” “Targets Set,” and selected “Human Protein Targets Only (v2010, 2241);” the remaining options had the default value, and clicked “submit.” Finally, it was compared with target protein from the CTD database.

## 3. Results

### 3.1. Compounds, Corresponding Targets, and Genes of Coicis Semen

**Figure 2:**
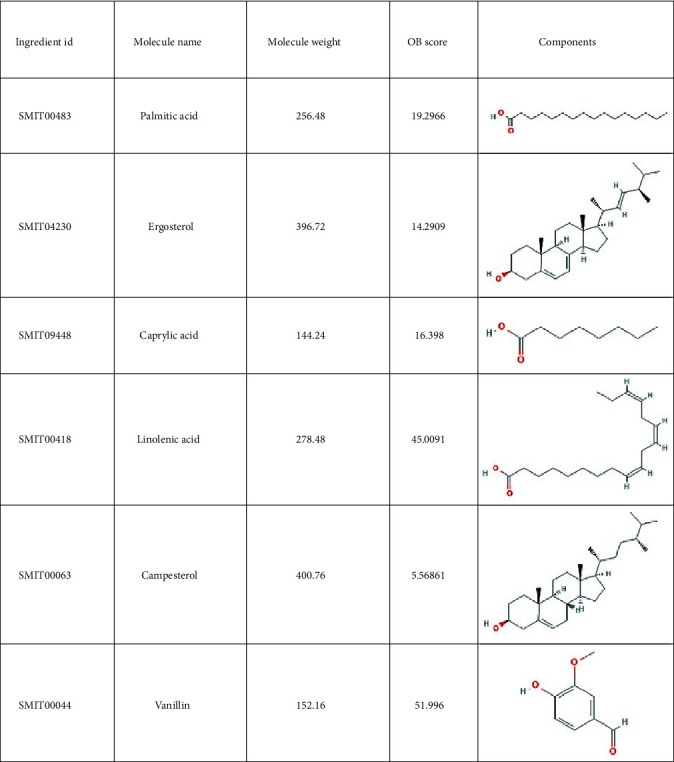
Small molecule components in Coicis Semen.

**Figure 3:**
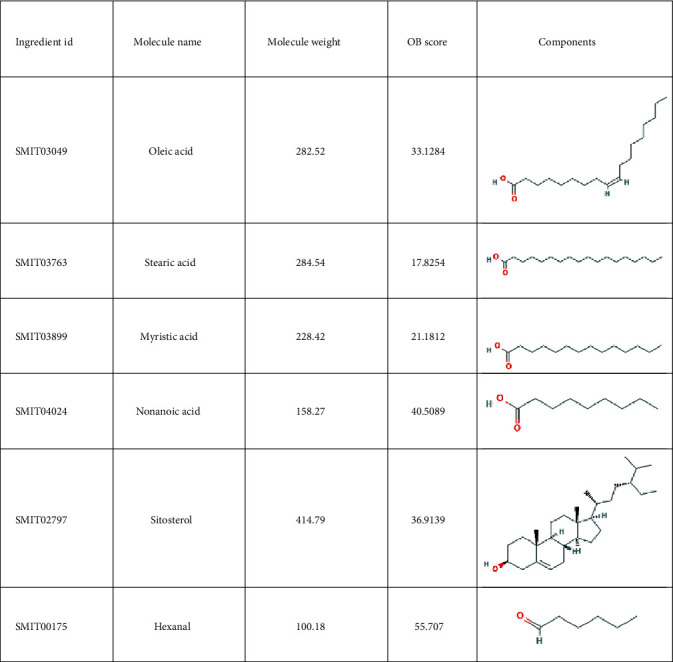
Small molecule components in Coicis Semen.

**Figure 4:**
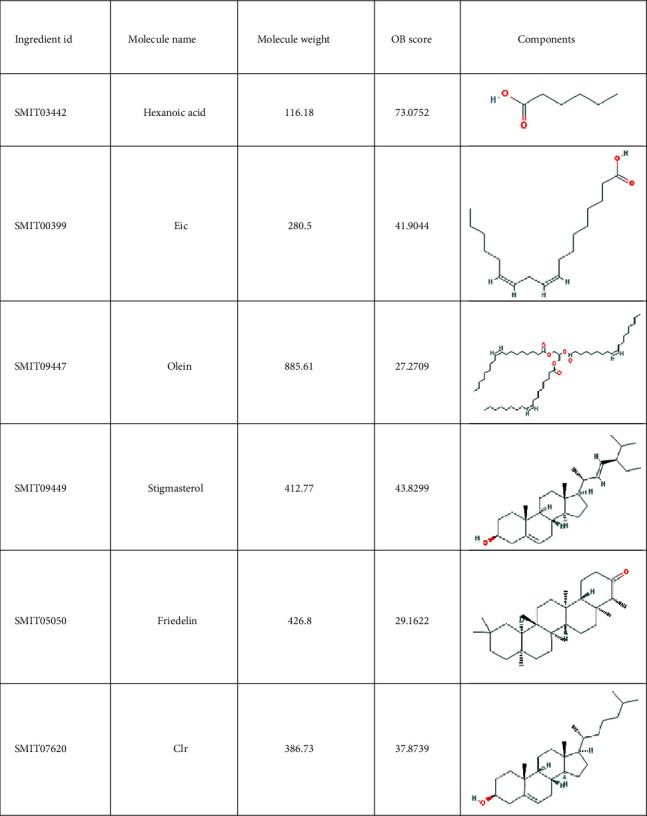
Small molecule components in Coicis Semen.

**Figure 5:**
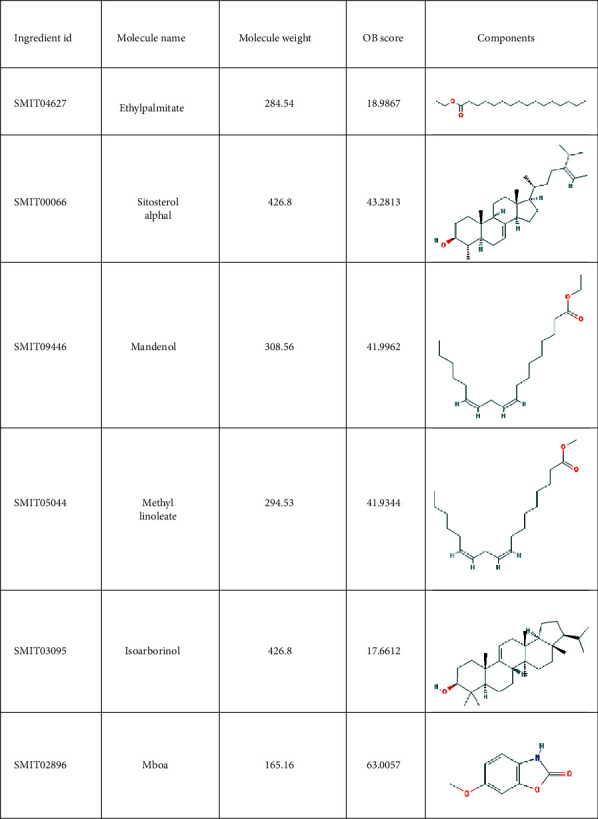
Small molecule components in Coicis Semen.

**Figure 6:**
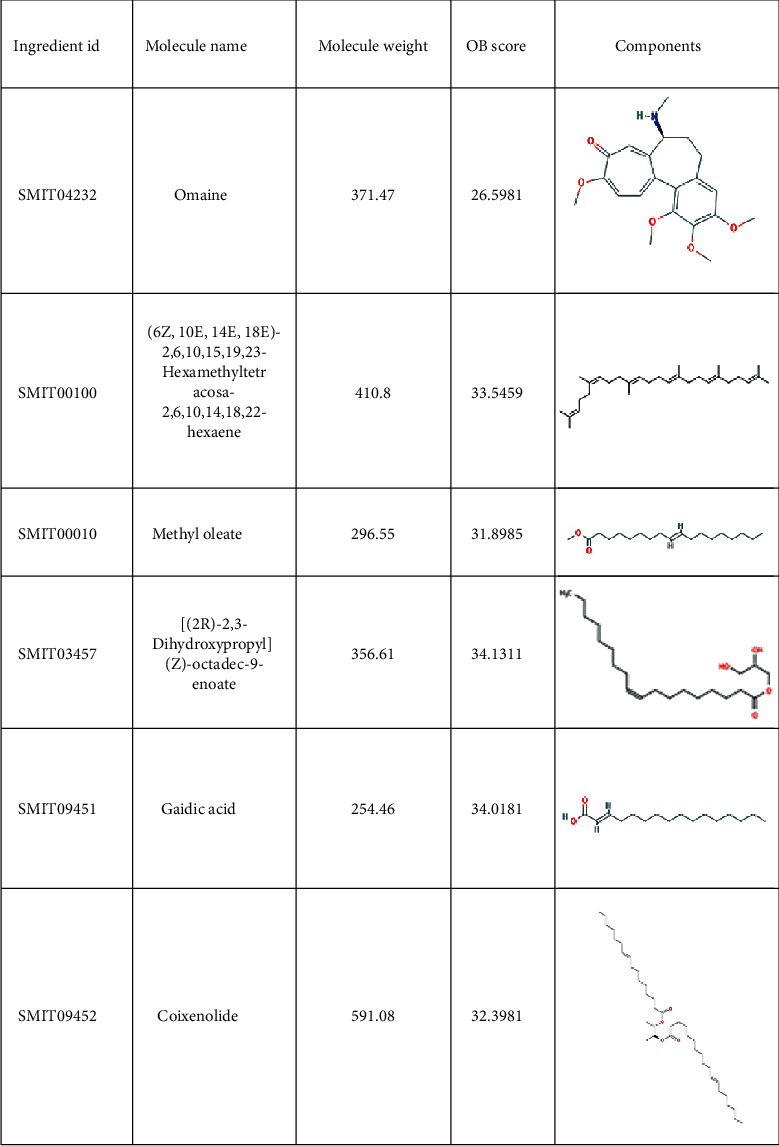
Small molecule components in Coicis Semen.

**Figure 7:**
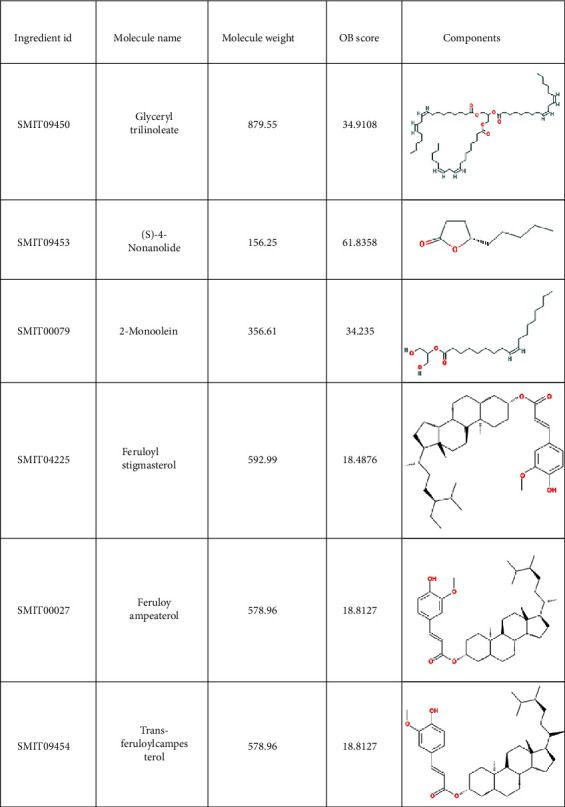
Small molecule components in Coicis Semen.

**Figure 8:**
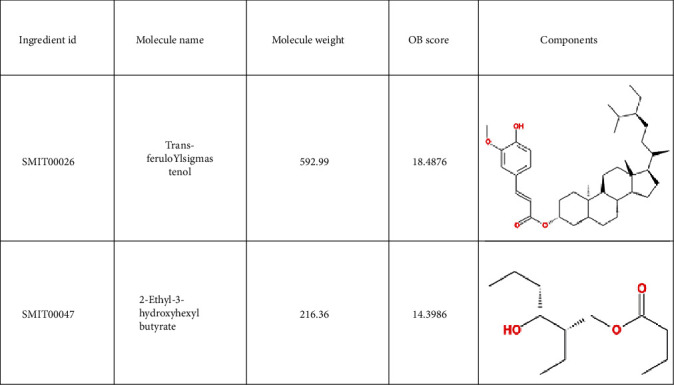
Small molecule components in Coicis Semen.

A total of 38 compounds of CS were obtained, of which 22 with oral bioavailability above 30 (see Figures 2–8). One hundred six target proteins were related to CS, 32282 liver cancer genes were obtained, and a total of 103 genes were shared by both (see Supplementary Data Table 1).

### 3.2. Coicis Semen-Hepatocarcinoma GO Enrichment and KEGG Enrichment Results

**Figure 9:**
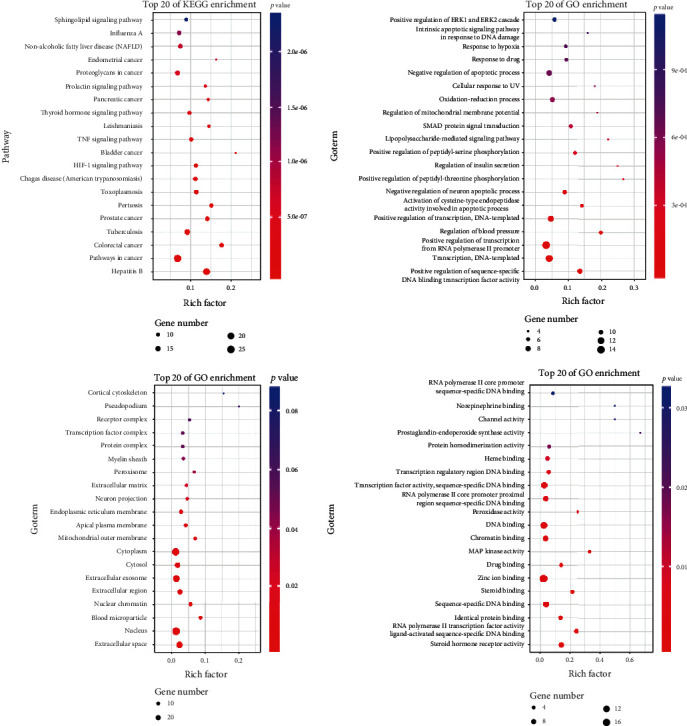
Gene enrichment results of “Coicis Semen-liver cancer.” Note: upper left KEGG pathways; upper right GO BP; lower left GO CC; lower right GO MF.

The results of the enrichment analysis are shown in Figure 9. The biological process (BP) analysis has a higher enrichment of positive regulation of sequence-specific DNA binding transcription factor activity, transcription, DNA-templated, and positive regulation of transcription from RNA polymerase II promoter. The cellular component (CC) analysis has a higher enrichment of extracellular space, nucleus, and blood microparticles. Molecular function (MF) analysis has a higher enrichment of steroid hormone receptor activity, RNA polymerase II transcription factor activity, ligand-activated sequence-specific DNA binding, and identification of protein binding. The results of KEGG enrichment analysis showed that “pathways in cancer” was the most closely related to the function of CS in treating liver cancer. The top 20 enrichment results are shown in Supplementary Data Table 2.

### 3.3. Potential Target Interaction Network and Analysis

**Figure 10:**
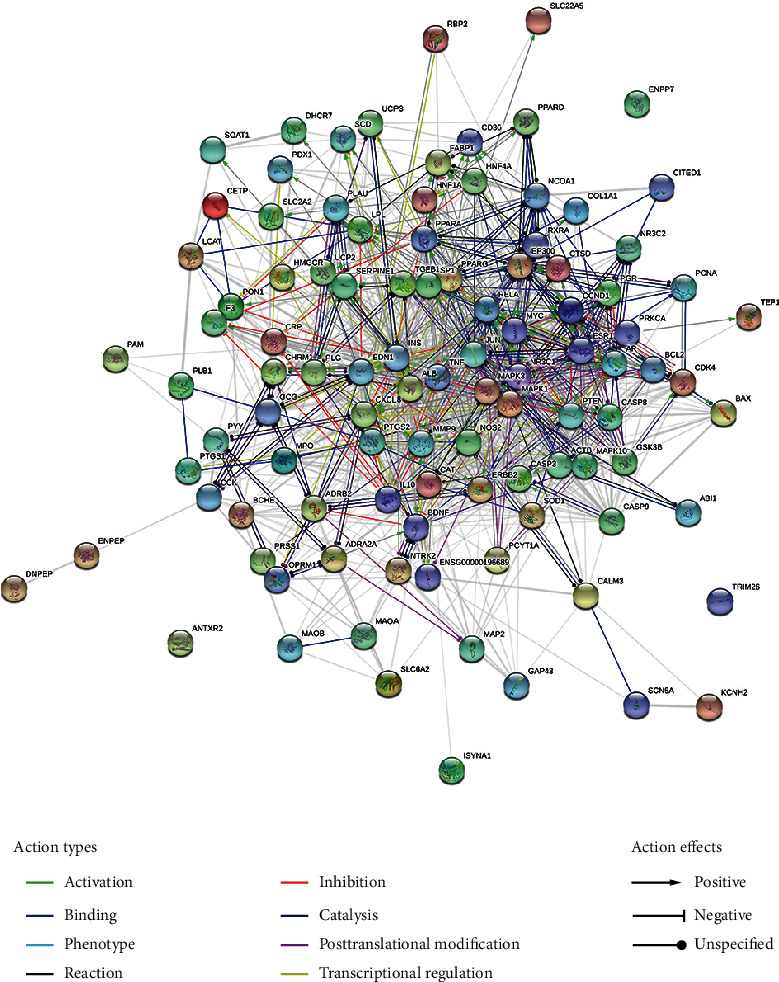
Interaction relationship between target proteins of Coicis Semen in treating liver cancer (based on String analysis). Note: the common target protein of “Coicis Semen-liver cancer;” PPI enrichment *p* value: <1.0*e*-16.

**Figure 11:**
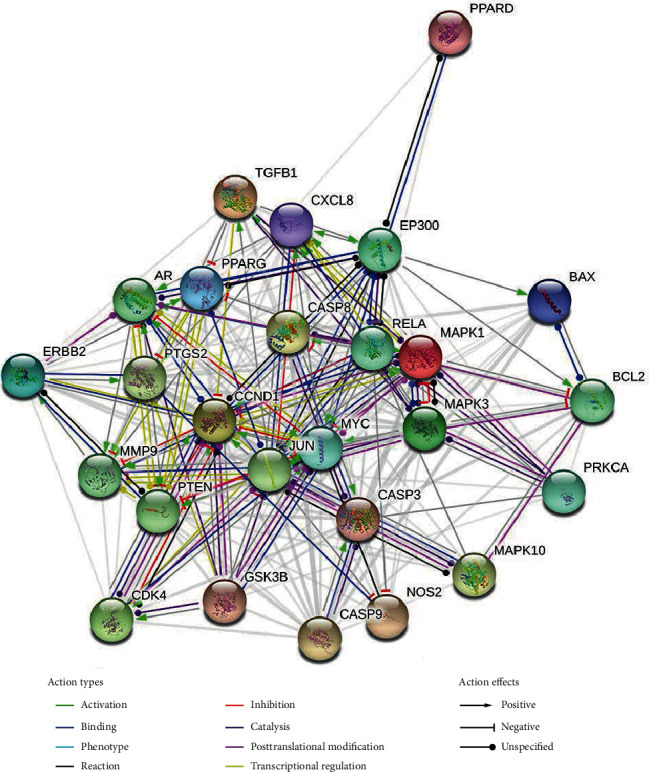
Interaction relationship between target proteins of Coicis Semen in treating liver cancer (based on String analysis). Note: the target protein enriched to “pathways in caner.” PPI enrichment *p* value: <1.0*e*-16.

**Figure 12:**
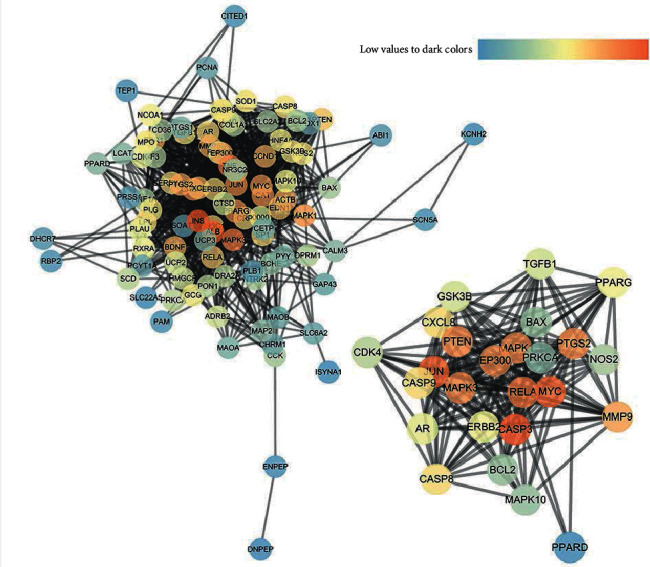
Interaction network of potential protein of Coicis Semen in treating liver cancer (based on Cytoscape composition results). Note: on the left is the common target protein of “Coicis Semen -liver cancer;” on the right is the target protein enriched to “pathways in cancer.”

As shown in Figures 10–12, the target interaction network diagram of “Coicis Semen-Liver Cancer” contains a total of 102 circular nodes, representing all predicted targets; 1,096 edges represent the correlation between the targets; the brighter and deeper the color of the nodes, the greater the degree value. The targets enriched to “pathways in cancer” are sorted according to the degree value, as shown in Supplementary Data Table 3. The number of top 5% is 1 in total, which is CASP3 [23], and the number is Cytoscape Degree in brackets. These targets are most closely related to “pathways in cancer,” which was the core target.

### 3.4. Relationship Network between Coicis Semen and CASP3 Protein

**Figure 13:**
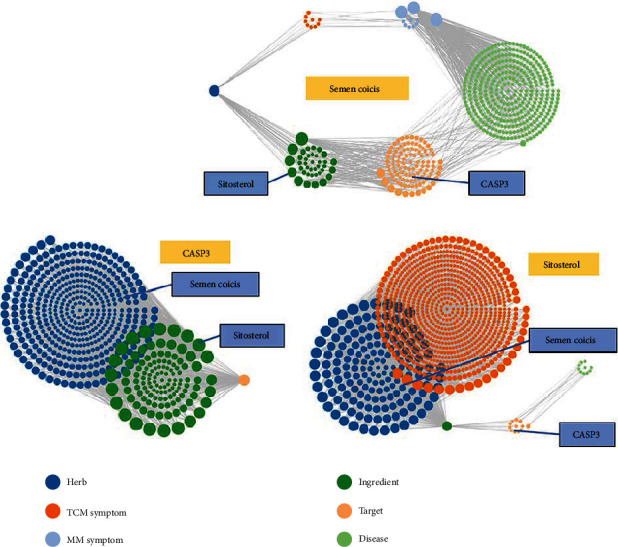
The relationship network between Coicis Semen, sitosterol, and CASP3 in the SymMap database.

According to the database results (Figure 13), it was found that “Sitosterol” is the bridge between CS and CASP3, that is, “Coicis Semen-Sitosterol-CASP3” is the relationship network between Coicis Semen and CASP3 protein.

### 3.5. Comparison of Targets of Sitosterol and Sorafenib in the Treatment of Liver Cancer

**Figure 14:**
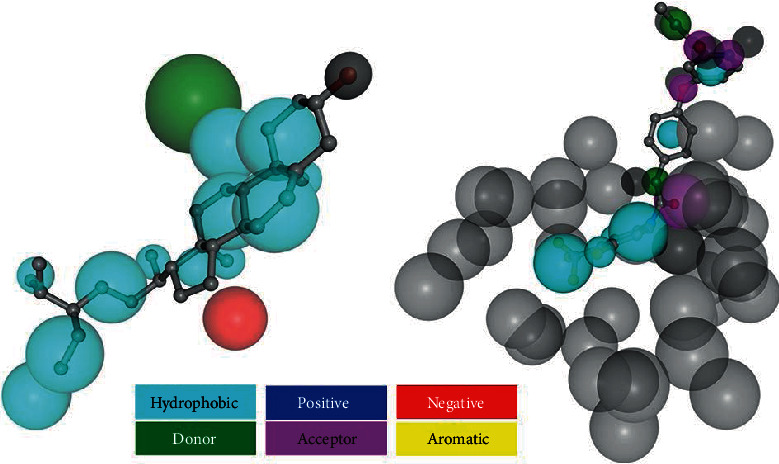
Interaction results with CASP3. Note: sitosterol is on the left and sorafenib is on the right.

As shown in Figure 14, sitosterol obtained 212 targets, while sorafenib got 300 targets. Among them, there are 137 targets of sitosterol in treating liver cancer, and the remaining 75 have nothing to do with liver cancer. There are 195 targets of sorafenib in treating liver cancer, and 105 have nothing to do with liver cancer. The two small molecules share 122 common targets for liver cancer, as shown in Supplementary Data Tables 4 to 6 of the Supplementary Data. According to the classification of protein function, two molecules fishing with more than ten proteins include TRANSFERASE and HYDROLASE. The number of sitosterol was 24 and 15, and the number of sorafenib was 31 and 24 (see Supplementary Data Table 7). The binding of two molecules and the casp3 protein is shown in Figure 14 and Table 2.

## 4. Discussion

At present, it is generally believed that liver cirrhosis is the leading risk factor for liver cancer. But several pieces of evidence show that the occurrence of liver cancer involves a variety of primary diseases such as hepatitis virus, aflatoxin, obesity, type 2 diabetes, nonalcoholic fatty liver, and metabolic syndrome [26–28]. Due to the multifactorial and complex pathophysiology of liver cancer, various treatment methods for tumors have side effects in varying degrees, such as skin toxicity events for targeted therapy [29]. Chinese herbal medicine is regarded as a natural library of compounds due to its multiple targets [30, 31] and thus has become a treatment alternative for various tumor supplements [32–34]. It is explored from a holistic perspective by a systematic approach such as network pharmacology, the relationship between drugs and diseases can provide new ideas for the research of traditional Chinese medicine.

Among the top 20 pathways enriched by the KEGG pathway, excluding tuberculosis, whooping cough, and other diseases that are not directly related to liver cancer, different pathways can be divided into the following categories: (a) cancer-related mechanisms: pathways in cancer, Proteoglycans in cancer; (b) precancerous lesions of the liver: hepatitis B, nonalcoholic fatty liver disease (NAFLD); (c) other cancers: colorectal cancer, prostate cancer, bladder cancer, endometrial cancer, pancreatic cancer; (d) other biological mechanisms: HIF-1 signaling pathway, TNF signaling pathway, thyroid hormone signaling pathway, prolactin signaling pathway, and sphingolipid signaling pathway. Hepatitis B [35] and fatty liver [36] are recognized causes of precancerous lesions of the liver. In contrast, pancreatic cancer, bladder cancer, etc. can cause metastatic liver malignancies in the liver through hematogenous metastasis. This study found that CS may have a particular therapeutic effect on the related diseases. If an experiment verifies these findings, CS can be used as a supplementary treatment plan for precancerous lesions of liver cancer.

Hypoxia-inducible factor 1 (HIF-1) is a transcription factor that acts as a major regulator of oxygen homeostasis. Various studies have demonstrated [37] that the upregulation of CD147 can protect liver cancer cells from apoptosis by glycolysis, switching through HIF-1 and MCT-4 under hypoxic conditions. As a significant cytokine, tumor necrosis factor (TNF) can induce a variety of intracellular signaling pathways, including apoptosis and cell survival, as well as inflammation and immunity. Adipose tissue is considered to be an endocrine organ that secretes proinflammatory cytokines such as tumor necrosis factor, and they are closely related to the progression of hepatocellular carcinoma (HCC) [38]. Thyroid hormones (THs) are significant regulators of growth, development, and metabolism. For example, thyroid hormone receptor-interacting agent A 13A (TRIP13) [39] may act as a tumor promoter during the development of liver cancer. The deletion of TRIP13 may damage the repair process of nonhomologous end joining A (NHEJ) in hepatocellular carcinoma A (HCC). Prolactin (PRL) [40] is a peptide hormone known to be involved in a variety of biological functions, including osmoregulation, lactation, reproduction, growth and development, endocrinology and metabolism, brain and behavior, and immune regulation. Prolactin is closely related to breast cancer, while liver metastases often occur in breast cancer patients [41]. Sphingomyelin (SM) and its metabolites have secondary messenger functions in a variety of cellular signaling pathways, and their signaling could be a useful target for preventing obesity-related breast cancer metastasis, such as liver cancer. In summary, it can be seen that CS may play a role in regulating oxygen homeostasis, anti-inflammatory, interfering with DNA replication, and inhibiting cancer cells from metastasizing to the liver [42].

**Figure 15:**
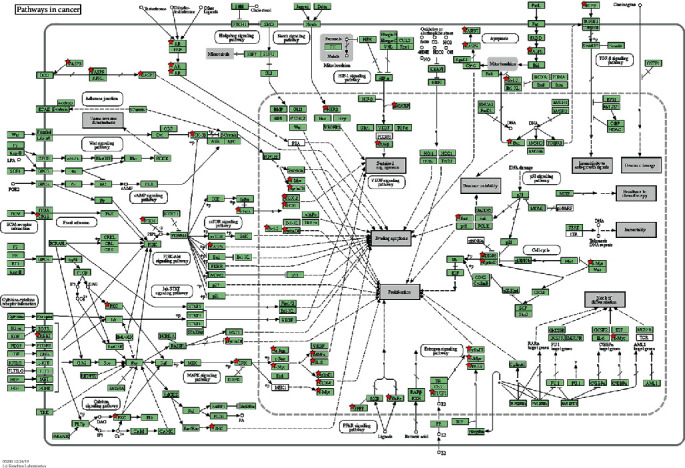
Coicis Semen is enriched in Pathways in cancer. Note: the red pentagram is a protein mapped by Coicis Semen.

The research shows that CASP3 could be a core protein of interaction network in CS for liver cancer, and sitosterol can be precisely combined with the protein, and eventually enriched in “Pathways in cancer,” as shown in Figure 15. CASP3 is a member of the caspase protein. Text mining tool Polysearch 2 (http://polysearch.cs.ualberta.ca/index) [43] and gene retrieval tool PubMed Gene (https://www.ncbi.nlm.nih.gov/gene/) [44] were applied to match liver cancer-related proteins. The results showed that CASP3 protein was one of the characteristic proteins of liver cancer. Sitosterol is a kind of phytosterol, which has been proven to have specific therapeutic effects on different tumors [45]. As a classic marker of apoptosis, it is activated by death ligands [46, 47] and interferes with normal apoptosis of cells. Sitosterol is widely present in natural plants. Studies have shown that I2-sitosterol [45] can significantly hinder the expansion of transplanted tumors, increasing the spleen cell proliferation and cytotoxic T lymphocyte (CTL) activity of model animals, and enhancing host macrophage lysosomal activity and antioxidant cell activity and inhibiting lipid peroxidation. Therefore, sitosterol may be competitively combined with CASP3 to inhibit cancer development. Besides, compared to sorafenib, sitosterol has fewer off-target genes in liver cancer and has a higher degree of binding to CASP3 protein.

## 5. Conclusion

In summary, this study builds an interaction network, based on the network pharmacology approach to predicting the interactions between compounds in Coicis Semen that could be related to the treatment of liver cancer and target genes. The results indicate that Coicis Semen may play a role in treating liver cancer by acting on CASP3 protein. Based on the systems biology analysis, it is speculated that Coicis Semen may not only participate in multiple mechanisms of action to treat liver cancer synergistically but may also be involved in factors that reduce the incidence of liver cancer. Therefore, it is considered that Coicis Semen can be a candidate drug in the treatment of liver cancer. However, the results obtained from systems biology analysis still need to be verified by pharmacological methods and omics techniques.

## Figures and Tables

**Table 1:** Research report of Coicis Semen in treatment of liver cancer.

No.	Dosage	Mechanisms	Target	Literature
1	Norcantharidin (NCTD) + Coix lacryma-jobi seed oil (CLSO) combination	Cytotoxicity and apoptotic induction in human HepG2 and HepG2/ADM cells	Hepal-1 hepatoma-bearing mice	[12]
2	Cy5/gal(oct)-C-MEs	Asialoglycoprotein receptor-mediated endocytosis	HepG2 xenograft-bearing nude mice	[13]
3	But-gal-CMEs	Strong cytotoxicity and improved apoptosis induction	HepG2 cells model	[14]
4	Gal-C-MEs	Strong cytotoxicity enhanced cellular uptake and improved apoptosis induction	HepG2 cells model	[15]
5	Kanglaite	Induction of NF-*κ*B-mediated gene transcription in CD4 T cells	Tumor-bearing mice	[16]
6	Adlay extracts	Obvious proliferate inhibition	Human liver cancer cells	[17]
7	Injectable extract from the seed of Coix lacryma-jobi	The expression of caspase-8 was elevated and prolonged	HepG2 cells	[18]
8	Ethanolic extract of adlay bran	Suppress CYP enzyme activities and CYP protein expression in the liver and lungs	Sprague-Dawley rats	[19]
9	Kanglaite	Mediated through activation of the Fas/FasL pathway	HepG2	[20]

**Table 2:** Interaction results among sitosterol and sorafenib and CASP3.

	Sitosterol	Sorafenib
PDB ID	1RHR	1GFW
Target name	Caspase-3	Caspase-3
Number of features	8	3
Fit score	4.122	2.67
Normalized fit score	0.5153	0.8901
*z*'-score	1.6793	0.0851835
Hydrophobic	7	2
Positive	0	0
Negative	1	0
Donor	0	0
Acceptor	0	1
Aromatic	0	0

## Data Availability

All the data has been added.

## Supplementary Materials

Supplementary Materials Table 1 describes the gene symbol shared by Coicis Semen and liver cancer. GO & KEGG enrichment results of Coicis Semen and liver cancer (top 20) are presented in Table 2. Table 3 describes target proteins enriched in “pathways in cancer” (Coicis Semen). Common targets for treating liver cancer are given in Table 4. Table 5 describes sitosterol's unique targets for treating liver cancer. Sorafenib's unique targets for treating liver cancer are mentioned in Table 6. Table 7 shows the protein classification of sitosterol and sorafenib.
