# Supplementary material for: Regulatory Mechanisms of Coicis Semen on Bionetwork of Liver Cancer Based on Network Pharmacology
Source: Biomed Res Int. 2020 Nov 21;2020:5860704. doi: 10.1155/2020/5860704 (PMC7700039; doi:10.1155/2020/5860704)
Supplement: Supplementary Materials — Table 1 describes the gene symbol shared by Coicis Semen and liver cancer. GO & KEGG enrichment results of Coicis Semen and liver cancer (top 20) are presented in Table 2. Table 3 describes target proteins enriched in “pathways in cancer” (Coicis Semen). Common targets for treating liver cancer are given in Table 4. Table 5 describes sitosterol's unique targets for treating liver cancer. Sorafenib's unique targets for treating liver cancer are mentioned in Table 6. Table 7 shows the protein classification of sitosterol and sorafenib. [file 5860704.f1.docx]

**Regulatory mechanisms of Coicis Semen on bio-network of liver cancer -based on network pharmacology**

Bonan Liu^1^, Chen Bai^2,*^

Gene Symbol shared by Coicis Semen and Liver Cancer have been presented in Table 1. Table 2 describes top 20 GO & KEGG enrichment results of coicis semen and liver cancer. Target Proteins enriched in "Pahtways in cancer" (Coicis Semen) have been given in Table 3. Table 4 presents Common targets for treating liver cancer. Sitosterol's unique targets for treating liver cancer are mentioned in Table 5. Table 6 portrays sorafenib's unique targets for treating liver cancer. Table 7 presents protein classification of sitosterol and sorafenib.

Table 1 Gene Symbol shared by Coicis Semen and Liver Cancer

| Target id | Gene symbol | Gene name | P_value | FDR(BH) |
| --- | --- | --- | --- | --- |
| SMTT00062 | ACTB | Actin Beta | 0.003874 | 0.016701 |
| SMTT00103 | ADRA2A | adrenergic, alpha-2A-, receptor | 0.426074 | 0.458124 |
| SMTT00106 | ADRB2 | adrenergic, beta-2-, receptor, surface | 0.88752 | 0.901691 |
| SMTT00150 | ALB | Albumin | 0.005806 | 0.020412 |
| SMTT00214 | ANTXR2 | Anthrax Toxin Receptor 2 | 0.001939 | 0.01143 |
| SMTT00244 | AR | Androgen Receptor | 0.515198 | 0.547202 |
| SMTT00356 | BCHE | butyrylcholinesterase | 0.392366 | 0.424021 |
| SMTT00361 | BCL2 | BCL2, Apoptosis Regulator | 0.015481 | 0.033958 |
| SMTT00369 | BDNF | Brain Derived Neurotrophic Factor | 0.024924 | 0.045048 |
| SMTT00461 | CALM3 | Calmodulin 3 | 0.813353 | 0.833411 |
| SMTT00480 | CASP3 | Caspase 3 | 0.214502 | 0.242469 |
| SMTT00482 | CASP8 | Caspase 8 | 0.056607 | 0.079568 |
| SMTT00487 | CAT | Catalase | 0.094362 | 0.119055 |
| SMTT00521 | CCND1 | Cyclin D1 | 0.081943 | 0.10618 |
| SMTT00541 | CD36 | CD36 Molecule | 0.01158 | 0.028724 |
| SMTT00570 | CDK4 | Cyclin Dependent Kinase 4 | 0.056607 | 0.079568 |
| SMTT00612 | CETP | Cholesteryl Ester Transfer Protein | 0.003874 | 0.016701 |
| SMTT00636 | CHRM1 | cholinergic receptor muscarinic 1 | 0.664706 | 0.693045 |
| SMTT00721 | COL1A1 | Collagen Type I Alpha 1 Chain | 0.024924 | 0.045048 |
| SMTT00793 | CRP | C-reactive protein | 0.007734 | 0.023578 |
| SMTT00838 | CTSD | Cathepsin D | 0.031318 | 0.052242 |
| SMTT00851 | CXCL8 | C-X-C motif chemokine ligand 8 | 0.052932 | 0.075716 |
| SMTT00940 | DHCR7 | 7-dehydrocholesterol Reductase | 0.001939 | 0.01143 |
| SMTT01000 | DNPEP | aspartyl aminopeptidase | 0.001939 | 0.01143 |
| SMTT01057 | EDN1 | Endothelin 1 | 0.023029 | 0.042867 |
| SMTT01104 | ENPEP | glutamyl aminopeptidase | 0.003874 | 0.016701 |
| SMTT01108 | EP300 | E1A Binding Protein P300 | 0.005806 | 0.020412 |
| SMTT01124 | ERBB2 | Erb-b2 Receptor Tyrosine Kinase 2 | 0.01158 | 0.028724 |
| SMTT01141 | ESR1 | Estrogen Receptor 1 | 0.810685 | 0.830869 |
| SMTT01170 | F3 | coagulation factor III, tissue factor | 0.028705 | 0.049501 |
| SMTT01483 | GSK3B | glycogen synthase kinase 3 beta | 0.506005 | 0.537993 |
| SMTT01567 | HMGCR | 3-hydroxy-3-methylglutaryl-CoA reductase | 0.026816 | 0.047252 |
| SMTT01571 | HNF1A | HNF1 Homeobox A | 0.003874 | 0.016701 |
| SMTT01573 | HNF4A | Hepatocyte Nuclear Factor 4 Alpha | 0.005806 | 0.020412 |
| SMTT01671 | IGHG1 | immunoglobulin heavy constant gamma 1 | 0.00022 | 0.002533 |
| SMTT01682 | IL10 | Interleukin 10 | 0.038093 | 0.05985 |
| SMTT01724 | INS | Insulin | 0.019227 | 0.038405 |
| SMTT01744 | ISYNA1 | inositol-3-phosphate synthase 1 | 3.65E-05 | 0.00072 |
| SMTT01778 | JUN | Jun proto-oncogene, AP-1 transcription factor subunit | 0.000309 | 0.003204 |
| SMTT01803 | KCNH2 | Potassium Voltage-gated Channel Subfamily H Member 2 | 0.572519 | 0.603543 |
| SMTT01924 | LCAT | Lecithin-cholesterol Acyltransferase | 0.001939 | 0.01143 |
| SMTT01979 | LPL | Lipoprotein Lipase | 0.024924 | 0.045048 |
| SMTT02024 | MAOA | Monoamine Oxidase A | 0.315053 | 0.345502 |
| SMTT02025 | MAOB | monoamine oxidase B | 0.373405 | 0.404822 |
| SMTT02027 | MAP2 | microtubule associated protein 2 | 0.007734 | 0.023578 |
| SMTT02033 | MAPK1 | Mitogen-activated Protein Kinase 1 | 0.07657 | 0.100587 |
| SMTT02034 | MAPK10 | Mitogen-activated Protein Kinase 10 | 0.01158 | 0.028724 |
| SMTT02037 | MAPK3 | Mitogen-activated Protein Kinase 3 | 0.054771 | 0.077713 |
| SMTT02143 | MMP9 | Matrix Metallopeptidase 9 | 0.080156 | 0.104223 |
| SMTT02157 | MPO | Myeloperoxidase | 0.038093 | 0.05985 |
| SMTT02203 | MYC | MYC Proto-oncogene, BHLH Transcription Factor | 0.001155 | 0.008066 |
| SMTT02251 | NCOA1 | nuclear receptor coactivator 1 | 0.552564 | 0.584256 |
| SMTT02349 | NOS2 | nitric oxide synthase 2 | 0.381812 | 0.413288 |
| SMTT02379 | NR3C1 | Nuclear Receptor Subfamily 3 Group C Member 1 | 0.423809 | 0.455793 |
| SMTT02380 | NR3C2 | Nuclear Receptor Subfamily 3 Group C Member 2 | 0.024071 | 0.044093 |
| SMTT02402 | NTRK2 | Neurotrophic Receptor Tyrosine Kinase 2 | 0.003874 | 0.016701 |
| SMTT02430 | OPRM1 | opioid receptor mu 1 | 0.468547 | 0.50074 |
| SMTT02464 | PAM | peptidylglycine alpha-amidating monooxygenase | 0.001939 | 0.01143 |
| SMTT02490 | PCNA | Proliferating Cell Nuclear Antigen | 0.01541 | 0.033918 |
| SMTT02495 | PCYT1A | Phosphate Cytidylyltransferase 1, Choline, Alpha | 0.001939 | 0.01143 |
| SMTT02519 | PDX1 | Pancreatic And Duodenal Homeobox 1 | 0.001939 | 0.01143 |
| SMTT02550 | PGR | Progesterone Receptor | 0.000652 | 0.005333 |
| SMTT02604 | PLAU | Plasminogen Activator, Urokinase | 0.058439 | 0.08154 |
| SMTT02615 | PLG | Plasminogen | 0.007734 | 0.023578 |
| SMTT02668 | PON1 | Paraoxonase 1 | 0.000131 | 0.001758 |
| SMTT02679 | PPARA | peroxisome proliferator activated receptor alpha | 0.000328 | 0.003336 |
| SMTT02680 | PPARD | peroxisome proliferator activated receptor delta | 0.003874 | 0.016701 |
| SMTT02681 | PPARG | Peroxisome Proliferator Activated Receptor Gamma | 0.056607 | 0.079568 |
| SMTT02717 | PRKCA | protein kinase C alpha | 0.01732 | 0.036174 |
| SMTT02747 | PRSS1 | Serine Protease 1 | 0.446645 | 0.478827 |
| SMTT02769 | PTEN | Phosphatase And Tensin Homolog | 0.01541 | 0.033918 |
| SMTT02776 | PTGS1 | prostaglandin-endoperoxide synthase 1 | 0.002026 | 0.011811 |
| SMTT02777 | PTGS2 | prostaglandin-endoperoxide synthase 2 | 0.01649 | 0.03496 |
| SMTT02865 | RELA | RELA proto-oncogene, NF-kB subunit | 0.199012 | 0.226574 |
| SMTT02969 | RXRA | retinoid X receptor alpha | 0.015778 | 0.034162 |
| SMTT03005 | SCN5A | Sodium Voltage-gated Channel Alpha Subunit 5 | 0.884948 | 0.899387 |
| SMTT03048 | SERPINE1 | Serpin Family E Member 1 | 0.019227 | 0.038405 |
| SMTT03127 | SLC22A5 | Solute Carrier Family 22 Member 5 | 0.001939 | 0.01143 |
| SMTT03155 | SLC2A2 | Solute Carrier Family 2 Member 2 | 0.001939 | 0.01143 |
| SMTT03193 | SLC6A2 | Solute Carrier Family 6 Member 2 | 0.141464 | 0.167389 |
| SMTT03253 | SOAT1 | sterol O-acyltransferase 1 | 0.005806 | 0.020412 |
| SMTT03256 | SOD1 | Superoxide Dismutase 1 | 0.081943 | 0.10618 |
| SMTT03447 | TGFB1 | Transforming Growth Factor Beta 1 | 0.049243 | 0.071681 |
| SMTT03502 | TNF | tumor necrosis factor | 0.023709 | 0.04372 |
| SMTT03590 | TRPV1 | transient receptor potential cation channel subfamily V member 1 | 5.47E-05 | 0.000964 |
| SMTT03662 | UCP2 | Uncoupling Protein 2 | 2.19E-05 | 0.000496 |
| SMTT03844 | CCK | Cholecystokinin | 0.005806 | 0.020412 |
| SMTT03877 | BAX | Q07812 | 0.11871 | 0.144304 |
| SMTT03918 | CASP9 | Caspase 9 | 0.083727 | 0.108066 |
| SMTT03934 | GAP43 | P17677 | 0.009659 | 0.026331 |
| SMTT03938 | GCG | Glucagon | 0.005806 | 0.020412 |
| SMTT03952 | FABP1 | P07148 | 3.66E-06 | 0.000131 |
| SMTT03993 | SCD | Stearoyl-CoA Desaturase | 0.001939 | 0.01143 |
| SMTT04023 | RBP2 | Retinol Binding Protein 2 | 3.66E-06 | 0.000131 |
| SMTT04030 | CITED1 | Q99966 | 0.001939 | 0.01143 |
| SMTT04047 | PYY | Peptide YY | 0.001939 | 0.01143 |
| SMTT04068 | UCP3 | Uncoupling Protein 3 | 0.003874 | 0.016701 |
| SMTT04076 | TEP1 | Q99973 | 0.028705 | 0.049501 |
| SMTT04088 | TRIM26 | Q12899 | 0.001939 | 0.01143 |
| SMTT04091 | SP1 | Sp1 Transcription Factor | 0.003874 | 0.016701 |
| SMTT04128 | ABI1 | Abl Interactor 1 | 0.001939 | 0.01143 |
| SMTT04237 | PLB1 | Phospholipase B1 | 7.33E-05 | 0.00118 |
| SMTT04239 | ENPP7 | Q6UWV6 | 0.003874 | 0.016701 |

Table 2 GO&KEGG Enrichment Results of Coicis Semen and Liver Cancer (top 20)

| Category | Name | Count | Pop Hits | Pvalue | Genes |
| --- | --- | --- | --- | --- | --- |
| KEGG_PATHWAY | Hepatitis B | 21 | 151 | 2.76E-17 | PRKCA, TNF, RELA, MMP9, CXCL8, MAPK10, CDK4, PTEN, TGFB1, MAPK1, CASP3, CCND1, EP300, CASP9, BAX, BCL2, JUN, CASP8, MAPK3, PCNA, MYC |
|  | Pathways in cancer | 26 | 384 | 4.12E-14 | PPARD, PTGS2, ERBB2, MMP9, PPARG, CXCL8, PTEN, TGFB1, CASP3, CASP9, BCL2, CASP8, NOS2, MYC, PRKCA, AR, RELA, MAPK10, CDK4, MAPK1, CCND1, EP300, GSK3B, JUN, BAX, MAPK3 |
|  | Colorectal cancer | 12 | 68 | 8.10E-11 | MAPK1, CASP3, CCND1, CASP9, JUN, BCL2, GSK3B, BAX, MAPK3, MAPK10, MYC, TGFB1 |
|  | Tuberculosis | 16 | 174 | 2.37E-10 | TNF, RELA, MAPK10, TGFB1, IL10, MAPK1, CASP3, EP300, CASP9, BCL2, BAX, MAPK3, CASP8, CALM3, CTSD, NOS2 |
|  | Prostate cancer | 12 | 85 | 9.79E-10 | MAPK1, AR, CCND1, EP300, CASP9, INS, BCL2, RELA, GSK3B, ERBB2, MAPK3, PTEN |
|  | Pertussis | 11 | 73 | 3.40E-09 | MAPK1, CASP3, TNF, JUN, RELA, MAPK3, CXCL8, CALM3, MAPK10, NOS2, IL10 |
|  | Toxoplasmosis | 12 | 105 | 9.67E-09 | MAPK1, CASP3, TNF, CASP9, BCL2, RELA, MAPK3, CASP8, MAPK10, NOS2, TGFB1, IL10 |
|  | Chagas disease (American trypanosomiasis) | 12 | 107 | 1.18E-08 | MAPK1, TNF, JUN, RELA, MAPK3, CASP8, SERPINE1, CXCL8, MAPK10, NOS2, TGFB1, IL10 |
|  | HIF-1 signaling pathway | 11 | 97 | 5.63E-08 | PRKCA, MAPK1, EP300, INS, BCL2, RELA, ERBB2, MAPK3, EDN1, SERPINE1, NOS2 |
|  | Bladder cancer | 8 | 38 | 1.07E-07 | MAPK1, CCND1, MMP9, ERBB2, MAPK3, CXCL8, CDK4, MYC |
|  | TNF signaling pathway | 11 | 108 | 1.58E-07 | MAPK1, CASP3, TNF, PTGS2, JUN, MMP9, RELA, MAPK3, EDN1, CASP8, MAPK10 |
|  | Leishmaniasis | 9 | 62 | 2.27E-07 | MAPK1, TNF, PTGS2, JUN, RELA, MAPK3, NOS2, TGFB1, IL10 |
|  | Thyroid hormone signaling pathway | 11 | 113 | 2.43E-07 | ACTB, PRKCA, MAPK1, CCND1, NCOA1, EP300, CASP9, GSK3B, MAPK3, ESR1, MYC |
|  | Pancreatic cancer | 9 | 63 | 2.58E-07 | MAPK1, CCND1, CASP9, RELA, ERBB2, MAPK3, MAPK10, CDK4, TGFB1 |
|  | Prolactin signaling pathway | 9 | 66 | 3.72E-07 | MAPK1, CCND1, INS, RELA, SLC2A2, GSK3B, MAPK3, ESR1, MAPK10 |
|  | Proteoglycans in cancer | 13 | 190 | 5.68E-07 | PRKCA, ACTB, TNF, ERBB2, MMP9, ESR1, TGFB1, MAPK1, CCND1, CASP3, MAPK3, MYC, PLAU |
|  | Endometrial cancer | 8 | 49 | 6.66E-07 | MAPK1, CCND1, CASP9, GSK3B, ERBB2, MAPK3, MYC, PTEN |
|  | Non-alcoholic fatty liver disease (NAFLD) | 12 | 160 | 7.60E-07 | PPARA, CASP3, TNF, INS, JUN, RELA, GSK3B, BAX, CASP8, CXCL8, MAPK10, TGFB1 |
|  | Influenza A | 12 | 167 | 1.17E-06 | ACTB, MAPK1, EP300, TNF, CASP9, JUN, RELA, GSK3B, MAPK3, CXCL8, MAPK10, PLG |
|  | Sphingolipid signaling pathway | 10 | 112 | 2.30E-06 | PRKCA, MAPK1, TNF, BCL2, RELA, BAX, MAPK3, CTSD, MAPK10, PTEN |
| GOTERM_BP_DIRECT | positive regulation of sequence-specific DNA binding transcription factor activity | 7 | 51 | 2.01E-06 | EP300, JUN, PPARG, ESR1, TRIM26, PTEN, IL10 |
|  | transcription, DNA-templated | 12 | 290 | 9.82E-06 | PGR, PPARA, AR, PPARD, NCOA1, JUN, RXRA, MAPK3, PPARG, ESR1, NR3C1, MYC |
|  | positive regulation of transcription from RNA polymerase II promoter | 14 | 436 | 1.93E-05 | AR, TNF, RELA, PPARG, NR3C1, TGFB1, IL10, NCOA1, ADRB2, SP1, GSK3B, JUN, MAPK3, MYC |
|  | regulation of blood pressure | 5 | 25 | 3.27E-05 | PTGS2, PTGS1, PPARG, ENPEP, SOD1 |
|  | positive regulation of transcription, DNA-templated | 9 | 196 | 1.05E-04 | AR, PPARD, NCOA1, HNF1A, JUN, PPARG, ESR1, MYC, TGFB1 |
|  | activation of cysteine-type endopeptidase activity involved in apoptotic process | 5 | 35 | 1.28E-04 | TNF, CASP9, F3, PPARG, MYC |
|  | negative regulation of neuron apoptotic process | 6 | 68 | 1.49E-04 | BDNF, JUN, BCL2, BAX, SOD1, CITED1 |
|  | positive regulation of peptidyl-threonine phosphorylation | 4 | 15 | 1.72E-04 | GCG, MAPK1, GSK3B, TGFB1 |
|  | regulation of insulin secretion | 4 | 16 | 2.11E-04 | TNF, HNF1A, HNF4A, NOS2 |
|  | positive regulation of peptidyl-serine phosphorylation | 5 | 42 | 2.62E-04 | GCG, TNF, BCL2, GSK3B, TGFB1 |
|  | lipopolysaccharide-mediated signaling pathway | 4 | 18 | 3.04E-04 | MAPK1, TNF, MAPK3, TGFB1 |
|  | SMAD protein signal transduction | 5 | 47 | 4.06E-04 | HNF1A, HNF4A, JUN, TGFB1, CITED1 |
|  | regulation of mitochondrial membrane potential | 4 | 21 | 4.87E-04 | UCP2, BCL2, BAX, SOD1 |
|  | oxidation-reduction process | 7 | 137 | 5.50E-04 | HMGCR, SCD, MAOA, MAOB, CAT, NOS2, SOD1 |
|  | cellular response to UV | 4 | 22 | 5.61E-04 | EP300, CASP9, BAX, PCNA |
|  | negative regulation of apoptotic process | 8 | 196 | 6.45E-04 | GCG, CASP3, ALB, BCL2, GSK3B, CAT, MYC, PTEN |
|  | response to drug | 5 | 54 | 6.93E-04 | JUN, BCL2, CDK4, SOD1, MYC |
|  | response to hypoxia | 5 | 55 | 7.43E-04 | EP300, UCP2, EDN1, NOS2, PLAU |
|  | intrinsic apoptotic signaling pathway in response to DNA damage | 4 | 25 | 8.24E-04 | TNF, CASP9, BCL2, BAX |
|  | positive regulation of ERK1 and ERK2 cascade | 6 | 105 | 0.00111578 | GCG, OPRM1, JUN, MAPK3, PTEN, TGFB1 |
| GOTERM_CC_DIRECT | extracellular space | 18 | 762 | 1.36E-05 | LPL, TNF, MMP9, EDN1, CXCL8, SOD1, TGFB1, IL10, GCG, BCHE, INS, ALB, F3, SERPINE1, MPO, CTSD, PYY, PLAU |
|  | nucleus | 29 | 2096 | 2.38E-04 | PPARA, PPARD, HNF1A, ERBB2, PPARG, NR3C1, PDX1, CITED1, TGFB1, PGR, CASP3, CASP9, ALB, MYC, AR, RXRA, RELA, ESR1, SOD1, CDK4, NCOA1, ADRB2, HNF4A, SP1, GSK3B, JUN, PCNA, CALM3, MPO |
|  | blood microparticle | 5 | 58 | 6.60E-04 | ACTB, BCHE, ALB, TGFB1, DNPEP |
|  | nuclear chromatin | 6 | 107 | 8.30E-04 | ACTB, AR, PPARD, NCOA1, SP1, JUN |
|  | extracellular region | 10 | 407 | 0.001856808 | BDNF, CCK, LCAT, EDN1, CRP, PON1, PYY, TGFB1, PLG, DNPEP |
|  | extracellular exosome | 22 | 1590 | 0.002092521 | ACTB, PRKCA, PAM, MMP9, MAOB, PTGS1, ENPEP, SOD1, MAPK1, ALB, BAX, F3, LCAT, SERPINE1, MAPK3, PCNA, MPO, CALM3, FABP1, SLC22A5, CAT, PLAU |
|  | cytosol | 13 | 718 | 0.003522878 | ACTB, PRKCA, MAPK1, CASP3, BCL2, RELA, GSK3B, BAX, MAPK3, NOS2, CDK4, SOD1, CITED1 |
|  | cytoplasm | 26 | 2145 | 0.004108547 | HNF1A, RBP2, EDN1, ENPEP, NR3C1, PTEN, TGFB1, CASP3, BDNF, CASP9, SLC2A2, ADRA2A, AR, RELA, TRIM26, CDK4, SOD1, DNPEP, GCG, EP300, HNF4A, MAP2, PCNA, MPO, FABP1, GAP43 |
|  | endoplasmic reticulum membrane | 6 | 214 | 0.015773541 | SOAT1, HMGCR, BCL2, SCD, BAX, PCYT1A |
|  | neuron projection | 4 | 87 | 0.021843766 | OPRM1, SLC6A2, MAP2, PTEN |
|  | extracellular matrix | 4 | 90 | 0.023860346 | MMP9, F3, SERPINE1, SOD1 |
|  | peroxisome | 3 | 45 | 0.038043733 | CAT, NOS2, SOD1 |
|  | myelin sheath | 4 | 115 | 0.044436219 | ACTB, ALB, BCL2, SOD1 |
|  | protein complex | 4 | 123 | 0.052407838 | AR, HNF1A, ALB, SOD1 |
|  | transcription factor complex | 4 | 123 | 0.052407838 | EP300, HNF1A, JUN, CDK4 |
|  | receptor complex | 3 | 57 | 0.058200851 | ADRB2, ERBB2, ADRA2A |
|  | pseudopodium | 2 | 10 | 0.066518028 | MAPK1, MAPK3 |
|  | cortical cytoskeleton | 2 | 13 | 0.08560645 | ACTB, NOS2 |
|  | nucleoplasm | 11 | 919 | 0.097551634 | MAPK1, SP1, JUN, MAPK3, PCNA, FABP1, NR3C1, SOD1, MYC, PTEN, DNPEP |
|  | - | - | - | - | - |
| GOTERM_MF_DIRECT | steroid hormone receptor activity | 7 | 51 | 2.54E-06 | PGR, PPARA, PPARD, HNF4A, RXRA, PPARG, NR3C1 |
|  | RNA polymerase II transcription factor activity, ligand-activated sequence-specific DNA binding | 5 | 21 | 1.85E-05 | PPARA, AR, PPARD, HNF4A, PPARG |
|  | identical protein binding | 5 | 38 | 2.06E-04 | ACTB, TNF, INS, PPARG, SOD1 |
|  | sequence-specific DNA binding | 10 | 273 | 2.62E-04 | PGR, PPARA, AR, PPARD, RXRA, BCL2, PPARG, ESR1, PDX1, NR3C1 |
|  | steroid binding | 4 | 19 | 4.03E-04 | PGR, AR, ESR1, NR3C1 |
|  | zinc ion binding | 16 | 793 | 0.001082752 | PAM, PPARA, AR, PPARD, RXRA, MMP9, PPARG, ESR1, TRIM26, NR3C1, ENPEP, SOD1, DNPEP, PGR, EP300, HNF4A |
|  | drug binding | 4 | 29 | 0.001437674 | PPARA, PPARD, ALB, PPARG |
|  | MAP kinase activity | 3 | 9 | 0.002080975 | MAPK1, MAPK3, MAPK10 |
|  | chromatin binding | 8 | 233 | 0.002217037 | AR, NCOA1, JUN, RELA, PPARG, PCNA, FABP1, CITED1 |
|  | DNA binding | 11 | 471 | 0.003495814 | PPARA, AR, NCOA1, HNF1A, ALB, JUN, RELA, PPARG, ESR1, NR3C1, MYC |
|  | peroxidase activity | 3 | 12 | 0.003757654 | PTGS2, PTGS1, MPO |
|  | RNA polymerase II core promoter proximal region sequence-specific DNA binding | 7 | 195 | 0.004086704 | PGR, ACTB, PPARA, AR, SP1, JUN, NR3C1 |
|  | transcription factor activity, sequence-specific DNA binding | 10 | 406 | 0.004218765 | PGR, AR, HNF1A, RXRA, RELA, PPARG, ESR1, PDX1, NR3C1, MYC |
|  | transcription regulatory region DNA binding | 5 | 92 | 0.005685679 | AR, TNF, JUN, PPARG, CITED1 |
|  | heme binding | 5 | 106 | 0.009313798 | PTGS2, PTGS1, MPO, CAT, NOS2 |
|  | protein homodimerization activity | 4 | 70 | 0.017141276 | ADRB2, BCL2, BAX, ADRA2A |
|  | prostaglandin-endoperoxide synthase activity | 2 | 3 | 0.023173414 | PTGS2, PTGS1 |
|  | channel activity | 2 | 4 | 0.030779307 | BCL2, BAX |
|  | norepinephrine binding | 2 | 4 | 0.030779307 | ADRB2, ADRA2A |
|  | RNA polymerase II core promoter sequence-specific DNA binding | 3 | 36 | 0.031797855 | EP300, HNF1A, HNF4A |

Table 3 Target Proteins enriched in "Pahtways in cancer" (Coicis Semen)

| name | Degree | Average Shortest Path Length | Betweenness Centrality | Closeness Centrality | Clustering Coefficient |
| --- | --- | --- | --- | --- | --- |
| CASP3 | 23 | 1.04166667 | 0.03660298 | 0.96 | 0.68379447 |
| MYC | 22 | 1.08333333 | 0.02866072 | 0.92307692 | 0.71428571 |
| JUN | 22 | 1.08333333 | 0.02941411 | 0.92307692 | 0.70995671 |
| RELA | 21 | 1.125 | 0.04622083 | 0.88888889 | 0.68095238 |
| PTEN | 20 | 1.16666667 | 0.01649114 | 0.85714286 | 0.77368421 |
| PTGS2 | 20 | 1.16666667 | 0.03575011 | 0.85714286 | 0.73684211 |
| EP300 | 20 | 1.16666667 | 0.04788871 | 0.85714286 | 0.66842105 |
| MAPK3 | 20 | 1.16666667 | 0.02233264 | 0.85714286 | 0.74210526 |
| MAPK1 | 20 | 1.16666667 | 0.01972901 | 0.85714286 | 0.75789474 |
| MMP9 | 19 | 1.20833333 | 0.01248847 | 0.82758621 | 0.80701754 |
| CASP9 | 17 | 1.29166667 | 0.01345583 | 0.77419355 | 0.75735294 |
| CXCL8 | 17 | 1.29166667 | 0.00506786 | 0.77419355 | 0.88235294 |
| CASP8 | 17 | 1.29166667 | 0.01262032 | 0.77419355 | 0.77941176 |
| ERBB2 | 16 | 1.33333333 | 0.00473071 | 0.75 | 0.875 |
| PPARG | 16 | 1.33333333 | 0.007476 | 0.75 | 0.85 |
| AR | 15 | 1.375 | 0.0032994 | 0.72727273 | 0.8952381 |
| TGFB1 | 14 | 1.41666667 | 0.00124753 | 0.70588235 | 0.95604396 |
| GSK3B | 14 | 1.41666667 | 0.00835037 | 0.70588235 | 0.78021978 |
| CDK4 | 12 | 1.5 | 0.00192903 | 0.66666667 | 0.89393939 |
| NOS2 | 11 | 1.54166667 | 0 | 0.64864865 | 1 |
| BAX | 10 | 1.58333333 | 0.00237367 | 0.63157895 | 0.84444444 |
| BCL2 | 10 | 1.58333333 | 0.00557425 | 0.63157895 | 0.73333333 |
| MAPK10 | 10 | 1.58333333 | 0.00207327 | 0.63157895 | 0.88888889 |
| PRKCA | 9 | 1.625 | 0.00216507 | 0.61538462 | 0.86111111 |
| PPARD | 3 | 1.875 | 0 | 0.53333333 | 1 |

Table 4 Common targets for treating liver cancer

| Name | Class | Uniprot | PDB ID |
| --- | --- | --- | --- |
| Aldo-keto reductase family 1 member C2 | OXIDOREDUCTASE | AK1C2_HUMAN | 1j96_v |
| Bone morphogenetic protein 2 | HORMONE/GROWTH FACTOR | BMP2_HUMAN | 1reu_v |
| Thyroid hormone receptor beta | NONE | P10828 | 2pin_v |
| MAP kinase-activated protein kinase 2 | NONE | P49137 | 2jbp_v |
| Nuclear receptor ROR-alpha | LIPID BINDING PROTEIN | RORA_HUMAN | 1n83_v |
| Kinesin-like protein KIF11 | MOTOR PROTEIN,CELL CYCLE | KIF11_HUMAN | 2pg2_v |
| Collagenase 3 | MATRIX METALLOPROTEASE | MMP13_HUMAN | 830c_v |
| Estradiol 17-beta-dehydrogenase 1 | NONE | P14061 | 1qyx_v |
| Caspase-7 | HYDROLASE | CASP7_HUMAN | 1shj_v |
| SEC14-like protein 2 | NONE | O76054 | 1o6u_v |
| Bile salt sulfotransferase | NONE | Q06520 | 1j99_v |
| Estrogen receptor | NUCLEAR RECEPTOR | ESR1_HUMAN | 1qkt_v |
| cAMP-dependent protein kinase catalytic subunit alpha | NONE | P00517 | 1ydt_v |
| Bile acid receptor | TRANSCRIPTION | NR1H4_HUMAN | 1osh_v |
| cAMP-specific 3,5-cyclic phosphodiesterase 4B | HYDROLASE | PDE4B_HUMAN | 1xlz_v |
| Proto-oncogene tyrosine-protein kinase Src | TRANSFERASE | SRC_HUMAN | 2h8h_v |
| Mitogen-activated protein kinase 14 | NONE | Q16539 | 1zyj_v |
| Phenylethanolamine N-methyltransferase | TRANSFERASE | PNMT_HUMAN | 1n7i_v |
| Epidermal growth factor receptor | TRANSFERASE | EGFR_HUMAN | 2j5f_v |
| Annexin A5 | CALCIUM/PHOSPHOLIPID-BINDING | ANXA5_HUMAN | 1hak_v |
| Cell division protein kinase 2 | NONE | P24941 | 2c69_v |
| Sex hormone-binding globulin | TRANSPORT PROTEIN | SHBG_HUMAN | 1lho_v |
| Prothrombin | BLOOD CLOTTING | THRB_HUMAN | 1d4p_v |
| 3-phosphoinositide-dependent protein kinase 1 | TRANSFERASE | PDPK1_HUMAN | 2pe0_v |
| Troponin C, slow skeletal and cardiac muscles | NONE | P63316 | 1ih0_v |
| Serine/threonine-protein phosphatase 5 | NONE | P53041 | 1s95_v |
| Vascular endothelial growth factor receptor 2 | NONE | P35968 | 3cjg_v |
| S-methyl-5-thioadenosine phosphorylase | NONE | Q13126 | 1cg6_v |
| Serine/threonine-protein kinase Chk1 | TRANSFERASE | CHK1_HUMAN | 2ywp_v |
| Retinoic acid receptor RXR-alpha | TRANSCRIPTION | RXRA_HUMAN | 2acl_v |
| Oxysterols receptor LXR-alpha | DNA BINDING PROTEIN | NR1H3_HUMAN | 1uhl_v |
| Alpha-tocopherol transfer protein | NONE | P49638 | 1oiz_v |
| Glutathione S-transferase A1 | NONE | P08263 | 1gse_v |
| Adenosine kinase | NONE | P55263 | 2i6b_v |
| Insulin-like growth factor 1 receptor | NONE | P08069 | 3f5p_v |
| Steroid hormone receptor ERR1 | NONE | P11474 | 2pjl_v |
| Sulfotransferase family cytosolic 2B member 1 | NONE | O00204 | 1q22_v |
| Glutathione S-transferase P | TRANSFERASE | GSTP1_HUMAN | 19gs_v |
| Dipeptidyl peptidase 4 | HYDROLASE | DPP4_HUMAN | 2iiv_v |
| Nitric oxide synthase, endothelial | OXIDOREDUCTASE | NOS3_HUMAN | 1m9j_v |
| Hepatocyte nuclear factor 4-gamma | NONE | Q14541 | 1lv2_v |
| Receptor tyrosine-protein kinase erbB-4 | TRANSFERASE | ERBB4_HUMAN | 3bbt_v |
| Ephrin type-B receptor 4 | TRANSFERASE | EPHB4_HUMAN | 2vx0_v |
| E3 ubiquitin-protein ligase Mdm2 | LIGASE | MDM2_HUMAN | 1t4e_v |
| Proto-oncogene tyrosine-protein kinase LCK | TRANSFERASE | LCK_HUMAN | 1qpd_v |
| Oxysterols receptor LXR-beta | RECEPTOR | NR1H2_HUMAN | 1upw_v |
| Flavin reductase | NONE | P30043 | 1he3_v |
| Hepatocyte growth factor receptor | NONE | P08581 | 2rfn_v |
| cAMP-specific 3,5-cyclic phosphodiesterase 4D | HYDROLASE | PDE4D_HUMAN | 1xor_v |
| Gastrotropin | LIPID BINDING PROTEIN | FABP6_HUMAN | 1o1v_v |
| Fatty acid-binding protein, epidermal | LIPID-BINDING | FABP5_HUMAN | 1b56_v |
| Tyrosine-protein kinase ITK/TSK | NONE | Q08881 | 1sm2_v |
| cGMP-inhibited 3,5-cyclic phosphodiesterase B | HYDROLASE | PDE3B_HUMAN | 1so2_v |
| Peroxisome proliferator-activated receptor delta | NONE | Q03181 | 3dy6_v |
| Phospholipase A2, membrane associated | NONE | P14555 | 1dcy_v |
| Fibroblast growth factor receptor 2 | NONE | P21802 | 1oec_v |
| Nuclear receptor subfamily 1 group I member 3 | DNA BINDING PROTEIN | NR1I3_HUMAN | 1xvp_v |
| Peroxisome proliferator-activated receptor alpha | TRANSCRIPTION | PPARA_HUMAN | 1i7g_v |
| Alcohol dehydrogenase 1C | NONE | P00326 | 1u3w_v |
| Estrogen-related receptor gamma | NONE | P62508 | 2zas_v |
| Tyrosine-protein kinase JAK3 | TRANSFERASE | JAK3_HUMAN | 1yvj_v |
| Aldehyde dehydrogenase, mitochondrial | OXIDOREDUCTASE | ALDH2_HUMAN | 1of7_v |
| Estrogen receptor beta | TRANSCRIPTION | ESR2_HUMAN | 2i0g_v |
| Poly [ADP-ribose] polymerase 1 | NONE | P09874 | 1wok_v |
| Cyclin-A2 | TRANSFERASE | CCNA2_HUMAN | 2bpm_v |
| [Pyruvate dehydrogenase [lipoamide]] kinase isozyme 2, mitochondrial | TRANSFERASE | PDK2_HUMAN | 2bu5_v |
| Fatty acid-binding protein, brain | LIPID BINDING PROTEIN | FABP7_HUMAN | 1fe3_v |
| Serine/threonine-protein kinase PAK 7 | TRANSFERASE | PAK7_HUMAN | 2f57_v |
| Macrophage metalloelastase | HYDROLASE | MMP12_HUMAN | 1utt_v |
| Retinoic acid receptor gamma | GENE REGULATION | RARG_HUMAN | 1fcz_v |
| Ferrochelatase, mitochondrial | NONE | P22830 | 1hrk_v |
| cGMP-specific 3,5-cyclic phosphodiesterase | HYDROLASE | PDE5A_HUMAN | 1tbf_v |
| Neutrophil collagenase | HYDROLASE | MMP8_HUMAN | 1zvx_v |
| Cell division protein kinase 6 | CELL CYCLE/TRANSFERASE | CDK6_HUMAN | 1xo2_v |
| Cellular retinoic acid-binding protein 2 | NONE | P29373 | 1cbs_v |
| NAD(P)H dehydrogenase [quinone] 1 | FLAVOPROTEIN | NQO1_HUMAN | 1dxo_v |
| Interleukin-2 | CYTOKINE | IL2_HUMAN | 1m48_v |
| 72 kDa type IV collagenase | HYDROLASE/HYDROLASE INHIBITOR | MMP2_HUMAN | 1hov_v |
| Caspase-3 | HYDROLASE | CASP3_HUMAN | 1rhr_v |
| Superoxide dismutase [Mn], mitochondrial | NONE | P04179 | 1xil_v |
| Tyrosine-protein kinase ZAP-70 | TRANSFERASE | ZAP70_HUMAN | 1u59_v |
| Protein farnesyltransferase/geranylgeranyltransferase type-1 subunit alpha | TRANSFERASE | FNTA_HUMAN | 1sa4_v |
| Nuclear receptor subfamily 1 group I member 2 | TRANSCRIPTION | NR1I2_HUMAN | 2o9i_v |
| Insulin-like growth factor IA | NONE | P01343 | 1imx_v |
| Histo-blood group ABO system transferase | NONE | P16442 | 1r7t_v |
| Ganglioside GM2 activator | NONE | P17900 | 1tjj_v |
| Stromelysin-1 | HYDROLASE | MMP3_HUMAN | 1g4k_v |
| C-1-tetrahydrofolate synthase, cytoplasmic | NONE | P11586 | 1dia_v |
| Histamine N-methyltransferase | NONE | P50135 | 1jqe_v |
| Proto-oncogene tyrosine-protein kinase ABL1 | NONE | P00519 | 2hzi_v |
| RAC-beta serine/threonine-protein kinase | TRANSFERASE/INHIBITOR COMPLEX | AKT2_HUMAN | 2uw9_v |
| Basic fibroblast growth factor receptor 1 | TRANSFERASE | FGFR1_HUMAN | 3c4f_v |
| Retinoic acid receptor alpha | HORMONE/GROWTH FACTOR RECEPTOR | RARA_HUMAN | 1dkf_v |
| Protein-glutamine gamma-glutamyltransferase E | TRANSFERASE | TGM3_HUMAN | 1l9n_v |
| Retinoic acid receptor RXR-beta | NUCLEAR RECEPTOR | RXRB_HUMAN | 1h9u_v |
| Proactivator polypeptide | NONE | P07602 | 1n69_v |
| Dipeptidase 1 | HYDROLASE | DPEP1_HUMAN | 1itu_v |
| Vitamin D3 receptor | GENE REGULATION | VDR_HUMAN | 1s0z_v |
| Retinoic acid receptor beta | TRANSCRIPTION | RARB_HUMAN | 1xap_v |
| Serine/threonine-protein phosphatase PP1-gamma catalytic subunit | NONE | P36873 | 1jk7_v |
| Tyrosine-protein kinase JAK2 | TRANSFERASE | JAK2_HUMAN | 2b7a_v |
| Medium-chain specific acyl-CoA dehydrogenase, mitochondrial | ELECTRON TRANSFER | ACADM_HUMAN | 1egc_v |
| Deoxycytidine kinase | TRANSFERASE | DCK_HUMAN | 1p62_v |
| Cathepsin S | HYDROLASE | CATS_HUMAN | 2hhn_v |
| Mast/stem cell growth factor receptor | TRANSFERASE ACTIVATOR | KIT_HUMAN | 1t46_v |
| cAMP-dependent protein kinase, alpha-catalytic subunit | NONE | P00517 | 1svh_v |
| Tyrosine-protein kinase HCK | TYROSINE KINASE | HCK_HUMAN | 1qcf_v |
| Chymase | HYDROLASE | CMA1_HUMAN | 1t31_v |
| Heme oxygenase 1 | OXIDOREDUCTASE | HMOX1_HUMAN | 1s8c_v |
| Tyrosine-protein kinase CSK | TRANSFERASE | CSK_HUMAN | 3d7t_v |
| Glycogen phosphorylase, liver form | NONE | P06737 | 1l7x_v |
| Betaine--homocysteine S-methyltransferase 1 | NONE | Q93088 | 1lt8_v |
| Baculoviral IAP repeat-containing protein 4 | APOPTOSIS | XIAP_HUMAN | 1tfq_v |
| Bcl-2-like protein 1 | NONE | Q07817 | 2yxj_v |
| Chitotriosidase-1 | NONE | Q13231 | 1wb0_v |
| Caspase-1 | CYTOKINE | CASP1_HUMAN | 1ice_v |
| Histone-lysine N-methyltransferase SETD7 | TRANSFERASE | SETD7_HUMAN | 1mt6_v |
| Eukaryotic translation initiation factor 4E | NONE | P06730 | 2gpq_v |
| Glutathione S-transferase theta-2 | TRANSFERASE | GSTT2_HUMAN | 3ljr_v |
| Branched-chain-amino-acid aminotransferase, mitochondrial | NONE | O15382 | 1kt8_v |
| Insulin receptor | TRANSFERASE/SIGNALING PROTEIN | INSR_HUMAN | 2auh_v |
| Ornithine aminotransferase, mitochondrial | TRANSFERASE | OAT_HUMAN | 2can_v |

Table 5 Sitosterol's unique targets for treating liver cancer

| Name | Class | Uniprot | PDB ID |
| --- | --- | --- | --- |
| Steryl-sulfatase | HYDROLASE | STS_HUMAN | 1p49_v |
| Proto-oncogene serine/threonine-protein kinase Pim-1 | NONE | P11309 | 3bgp_v |
| Apolipoprotein A-II | LIPID TRANSPORT | APOA2_HUMAN | 1l6l_v |
| Histone deacetylase 8 | NONE | Q9BY41 | 3f0r_v |
| Beta-secretase 1 | HYDROLASE | BACE1_HUMAN | 2of0_v |
| Peroxisome proliferator-activated receptor gamma | NONE | P37231 | 3d6d_v |
| Glycogen synthase kinase-3 beta | NONE | P49841 | 3f7z_v |
| Growth factor receptor-bound protein 2 | PEPTIDE BINDING PROTEIN | GRB2_HUMAN | 1x0n_v |
| Cyclin-T1 | TRANSCRIPTION | CCNT1_HUMAN | 3blr_v |
| Heat shock protein HSP 90-beta | NONE | P08238 | 1uym_v |
| Serine/threonine-protein kinase 6 | NONE | O14965 | 3e5a_v |
| Transforming growth factor beta-2 | GROWTH FACTOR | TGFB2_HUMAN | 1tfg_v |
| Glutathione S-transferase Mu 1 | TRANSFERASE | GSTM1_HUMAN | 1xwk_v |
| FK506-binding protein 3 | ISOMERASE | FKBP3_HUMAN | 1pbk_v |
| Riboflavin kinase | NONE | Q969G6 | 1p4m_v |

Table 6 Sorafenib's unique targets for treating liver cancer

| Name | Class | Uniprot | PDB ID |
| --- | --- | --- | --- |
| Alpha-amylase 1 | NONE | P04745 | 1mfu_v |
| Salivary alpha-amylase | NONE | P04745 | 1mfv_v |
| Proto-oncogene serine/threonine-protein kinase Pim-1 | TRANSFERASE | PIM1_HUMAN | 1yxx_v |
| Peroxisome proliferator-activated receptor gamma | TRANSCRIPTION | PPARG_HUMAN | 1zgy_v |
| Beta-secretase 1 | NONE | P56817 | 3exo_v |
| Glutaminyl-peptide cyclotransferase | TRANSFERASE | QPCT_HUMAN | 2afu_v |
| Cytochrome P450 19A1 | NONE | P11511 | 3eqm_v |
| Putative ATP-dependent Clp protease proteolytic subunit, mitochondrial | HYDROLASE | CLPP_HUMAN | 1tg6_v |
| Reticulon-4 receptor | SIGNALING PROTEIN | RTN4R_HUMAN | 1ozn_v |
| Group 10 secretory phospholipase A2 | NONE | O15496 | 1le6_v |
| Macrophage migration inhibitory factor | IMMUNE SYSTEM | MIF_HUMAN | 1gcz_v |
| Ribosyldihydronicotinamide dehydrogenase [quinone] | NONE | P16083 | 3gam_v |
| Glutathione reductase, mitochondrial | NONE | P00390 | 1xan_v |
| Heat shock cognate 71 kDa protein | NONE | P11142 | 3fzk_v |
| Alcohol dehydrogenase 1B | OXIDOREDUCTASE | ADH1B_HUMAN | 1u3v_v |
| Bone morphogenetic protein 7 | HORMONE/GROWTH FACTOR | BMP7_HUMAN | 1m4u_v |
| Casein kinase II subunit alpha | NONE | P68400 | 3h30_v |
| Glycogen synthase kinase-3 beta | TRANSFERASE | GSK3B_HUMAN | 1q41_v |
| Nicotinamide mononucleotide adenylyltransferase 3 | NONE | Q96T66 | 1nup_v |
| Death-associated protein kinase 1 | TRANSFERASE | DAPK1_HUMAN | 1p4f_v |
| Tyrosyl-tRNA synthetase, cytoplasmic | NONE | P54577 | 1q11_v |
| Serine protease hepsin | NONE | P05981 | 1o5f_v |
| RAC-alpha serine/threonine-protein kinase | TRANSFERASE | AKT1_HUMAN | 3cqu_v |
| Histone deacetylase 8 | HYDROLASE | HDAC8_HUMAN | 1vkg_v |
| GTP-binding protein Rheb | NONE | Q15382 | 1xtq_v |
| Carbonyl reductase [NADPH] 1 | OXIDOREDUCTASE | CBR1_HUMAN | 1wma_v |
| Disintegrin and metalloproteinase domain-containing protein 17 | NONE | P78536 | 3edz_v |
| T-cell surface glycoprotein CD1a | IMMUNE SYSTEM | CD1A_HUMAN | 1onq_v |
| Protein-arginine deiminase type-4 | HYDROLASE | PADI4_HUMAN | 1wda_v |
| Hydroxyacyl-coenzyme A dehydrogenase, mitochondrial | NONE | Q16836 | 1m75_v |
| Fatty acid-binding protein, adipocyte | LIPID TRANSPORT | FABP4_HUMAN | 1tou_v |
| Transforming protein RhoA | NONE | P61586 | 1kmq_v |
| Serine/threonine-protein kinase PLK1 | NONE | P53350 | 2rku_v |
| Interferon-stimulated gene 20 kDa protein | HYDROLASE | ISG20_HUMAN | 1wlj_v |
| Tryptophan 5-hydroxylase 1 | OXIDOREDUCTASE | TPH1_HUMAN | 1mlw_v |
| Ras-related C3 botulinum toxin substrate 2 | NONE | P15153 | 1ds6_v |
| Cystathionine beta-synthase | NONE | P35520 | 1jbq_v |
| Galactokinase | TRANSFERASE | GALK1_HUMAN | 1wuu_v |
| B transferase | NONE | Q9NY01 | 1lzj_v |
| Lysozyme C | NONE | P61626 | 1rem_v |
| ADP-ribosyl cyclase 2 | HYDROLASE | BST1_HUMAN | 1isg_v |
| Rho GTPase-activating protein 1 | NONE | Q07960 | 1tx4_v |
| Thyroid hormone receptor beta-2 | NONE | P37243 | 1y0x_v |
| Bis(5-adenosyl)-triphosphatase | NONE | P49789 | 6fit_v |
| Matrilysin | METALLOPROTEASE | MMP7_HUMAN | 1mmq_v |
| Beta-hexosaminidase subunit beta | NONE | P07686 | 1np0_v |
| Matrix metalloproteinase-9 | HYDROLASE | MMP9_HUMAN | 1gkc_v |
| Copper transport protein ATOX1 | NONE | O00244 | 1fe0_v |
| Inositol monophosphatase | HYDROLASE | IMPA1_HUMAN | 1ima_v |
| Uridine-cytidine kinase 2 | NONE | Q9BZX2 | 1uej_v |
| Complement C1s subcomponent | HYDROLASE | C1S_HUMAN | 1elv_v |
| Suppressor of tumorigenicity protein 14 | HYDROLASE | ST14_HUMAN | 2gv7_v |
| Ephrin type-A receptor 2 | TRANSFERASE | EPHA2_HUMAN | 1mqb_v |
| Nitric oxide synthase, inducible | OXIDOREDUCTASE | NOS2_HUMAN | 2nsi_v |
| Cathepsin F | NONE | Q9UBX1 | 1m6d_v |
| Uridine 5-monophosphate synthase | NONE | P11172 | 3g3d_v |
| Thymidine kinase, cytosolic | NONE | P04183 | 1xbt_v |
| L-serine dehydratase | NONE | P20132 | 1p5j_v |
| Glutamate carboxypeptidase 2 | HYDROLASE | FOLH1_HUMAN | 2c6c_v |
| Phosphopantothenoylcysteine decarboxylase | NONE | Q96CD2 | 1qzu_v |
| Bifunctional 3-phosphoadenosine 5-phosphosulfate synthetase 1 | NONE | O43252 | 1x6v_v |
| Endoplasmic reticulum mannosyl-oligosaccharide 1,2-alpha-mannosidase | NONE | Q9UKM7 | 1x9d_v |
| Pleckstrin homology domain-containing family A member 4 | NONE | Q9H4M7 | 1upr_v |
| Adenine phosphoribosyltransferase | NONE | P07741 | 1ore_v |
| C-C motif chemokine 5 | ATTRACTANT | CCL5_HUMAN | 1u4l_v |
| Hydroxyacylglutathione hydrolase, mitochondrial | NONE | Q16775 | 1qh5_v |
| Glycolipid transfer protein | NONE | Q9NZD2 | 1sx6_v |
| Histidine triad nucleotide-binding protein 1 | PROTEIN KINASE INHIBITOR | HINT1_HUMAN | 1kpf_v |
| Hypoxanthine-guanine phosphoribosyltransferase | NONE | P00492 | 1hmp_v |
| Spermidine synthase | NONE | P19623 | 1zdz_v |
| Dual specificity protein kinase CLK1 | TRANSFERASE | CLK1_HUMAN | 1z57_v |
| Interstitial collagenase | NONE | P03956 | 1hfc_v |
| Deoxyuridine 5-triphosphate nucleotidohydrolase, mitochondrial | HYDROLASE | DUT_HUMAN | 1q5h_v |
| Eosinophil cationic protein | NONE | P12724 | 1dyt_v |

Table 7 Protein classification of sitosterol and sorafenib

| Class | sitosterol | sorafenib |
| --- | --- | --- |
| NONE | 53 | 88 |
| TRANSFERASE | 24 | 31 |
| HYDROLASE | 15 | 24 |
| TRANSCRIPTION | 8 | 9 |
| OXIDOREDUCTASE | 4 | 8 |
| LIPID BINDING PROTEIN | 3 | 3 |
| IMMUNE SYSTEM | - | 2 |
| NUCLEAR RECEPTOR | 2 | 2 |
| DNA BINDING PROTEIN | 2 | 2 |
| GENE REGULATION | 2 | 2 |
| CYTOKINE | 2 | 2 |
| HORMONE/GROWTH FACTOR | 1 | 2 |
| SIGNALING PROTEIN | - | 1 |
| LIPID TRANSPORT | 1 | 1 |
| METALLOPROTEASE | - | 1 |
| ATTRACTANT | - | 1 |
| PROTEIN KINASE INHIBITOR | - | 1 |
| MOTOR PROTEIN,CELL CYCLE | 1 | 1 |
| MATRIX METALLOPROTEASE | 1 | 1 |
| CALCIUM/PHOSPHOLIPID-BINDING | 1 | 1 |
| TRANSPORT PROTEIN | 1 | 1 |
| BLOOD CLOTTING | 1 | 1 |
| LIGASE | 1 | 1 |
| RECEPTOR | 1 | 1 |
| LIPID-BINDING | 1 | 1 |
| CELL CYCLE/TRANSFERASE | 1 | 1 |
| FLAVOPROTEIN | 1 | 1 |
| HYDROLASE/HYDROLASE INHIBITOR | 1 | 1 |
| TRANSFERASE/INHIBITOR COMPLEX | 1 | 1 |
| HORMONE/GROWTH FACTOR RECEPTOR | 1 | 1 |
| ELECTRON TRANSFER | 1 | 1 |
| TRANSFERASE ACTIVATOR | 1 | 1 |
| TYROSINE KINASE | 1 | 1 |
| APOPTOSIS | 1 | 1 |
| TRANSFERASE/SIGNALING PROTEIN | 1 | 1 |
| PEPTIDE BINDING PROTEIN | 1 | - |
| GROWTH FACTOR | 1 | - |
| ISOMERASE | 1 | - |
